# Tackling the Burden of Neurological Diseases in Canada with Virtual Care During the COVID-19 Pandemic and Beyond

**DOI:** 10.1017/cjn.2020.92

**Published:** 2020-05-12

**Authors:** Ramana Appireddy, Shirin Jalini, Garima Shukla, Lysa Boissé Lomax

**Affiliations:** Department of Medicine, Queen’s University, Kingston, ON, Canada

**Keywords:** Neurological practice, Telemedicine, General neurology, Digital health, eVisit, Virtual care

## The burden of neurological disease

Approximately 10% of Canadians are affected by neurological disorders based on Canada’s National Population Health Study of Neurological Conditions, and this number is projected to increase in the coming decades.^1^ Those with chronic neurological conditions have higher stress levels and a higher prevalence of self-diagnosed mood or anxiety disorders. Many suffer functional impairments with regard to cognition, mobility, dexterity, bowel, and bladder control. Taken together, these factors lead to significant impacts on quality of life. Neurological disease can also affect one’s ability to work or work productively, which can lead to financial insecurity.^1^ It can also result in a significant loss of the number of years (14–41 years based on condition) of healthy living. Based on the results from the Living with the Impact of a Neurological Condition (LINC) project, those with chronic neurological conditions use more health care services across the continuum compared to other chronic health conditions.^2^ In a study from British Columbia, physician service utilization is 1.4–5.6 times higher in people with chronic neurological conditions, as are the total direct health costs and out-of-pocket expenses for people affected by chronic neurological conditions.^1^ There are also limitations in health care services for this population with the physical environment cited as one of the significant barriers to the provision of adequate services. Thus, the burden of neurological diseases is not only shared by the individuals and families affected by it but also by the health care system.

There is an urgent need within our health care system to address the unmet needs of individuals suffering from neurological disease. Though it is not always easy to address the underlying biological mechanisms of these conditions, the health system performance can be optimized by adopting the Institute for Healthcare Improvement’s Triple Aim. This constitutes (1) reducing the per-capita cost of health care (out-of-pocket expenses and health system cost), (2) improving patient experiences of care (including quality and satisfaction), and (3) improving the health of populations.^3^ Virtual health care solutions are one way we can offer transformative changes to the practice of neurological ambulatory care in Canada, in order to meet some of the unmet needs of this challenging patient population.^1,4^

## Impact of COVID-19 on neurology outpatient care

Compounding the challenges mentioned above, the impact of COVID-19 on our health care is perceived by all. The COVID-19 crisis has resulted in temporary cancellation of elective clinics and all non-urgent/emergent clinical encounters in mid-late March across the country. A similar policy has resulted in cancellation of hundreds of outpatient encounters across all neurology clinics over the last few weeks. Some urgent and essential clinics like stroke prevention clinics and multiple sclerosis clinics were also indirectly affected by the COVID-19 crisis due to patients’ hesitation to come to a health care facility during the crisis. Their concern is genuine given the demographics (seniors in the stroke clinic) and patient characteristics (immunocompromised patients on multiple sclerosis medications). To meet the clinical needs of the patient care, the Division of Neurology at Queen’s University had completely transformed to a virtual care service to be able to provide the care. The transformation was significantly facilitated by the Ontario Ministry of Health’s decision to develop virtual care billing codes.^5^

## Virtual care

Virtual care has been defined as any interaction occurring remotely between patients and/or members of their circle of care, through any form of communication or information technology with the aim of facilitating or maximizing the quality and effectiveness of patient care.^6,7^ This can include secure messaging, secure email, or secure personal videoconferencing. Secure personal videoconferencing, also referred to as eVisit, is the use of personal internet-enabled devices like smartphones and tablets to videoconference with patients, with the goal to keep the patient at home or their preferred location. Digital health solutions are also adopted by the federal and provincial authorities as a key priority area of innovation to reduce health care costs.^8,9^ There is also growing demand by patients to have access to such services, as seen in a 2018 nationally representative survey of Canadians’ opinions on health care access.^10^

## Our experience with virtual care in neurology

We have successfully implemented eVisit pilot project in 2018 in the stroke clinic. The results of our pilot study demonstrated a very high degree of *patient satisfaction*, reduction in per capita health care costs, out of pocket expenses [mean(SD): $74.92(57.99) CND; median(IQR): $52.83(31.26–94.53) CND], health system costs (range between $23,832 to $28,584 dollars, just from the pilot), and statistically *significant reduction in wait times* for an eVisit follow-up compared to in-person follow-up.^11^ Physicians were able to assess patients more quickly via eVisit than via an in-person encounter, thus increasing the timely availability of health care.^12^ Adopting virtual care solutions can also result in a *significant reduction in costs*.^13^ The cost saving is a conservative estimate, and the actual figures are likely higher if other factors and social determinants like childcare, income status, other personal factors, and visit characteristics were accounted for. Our eVisit pilot project in neurology has made a provincial impact and is considered as an innovative model by the Ontario Telemedicine Network.^14^

Provided through the Ontario Telemedicine Network, eVisits were used exclusively for follow-up of clinical activities like the review of investigations, symptom management, therapeutic decisions, medication titration, other specialist consultations, patient counselling, and education. Following the successful experience from the pilot, the eVisits were scaled to other clinics in Neurology (sleep, epilepsy, and stroke). The eVisits were done from physicians’ offices using the office computers. The neurological examination is completed by following standard protocols adopted in other teleneurology settings like stroke and Parkinson’s disease.^15–17^ Patients are advised to record their blood pressure at home. The medication reconciliation is done by verifying the medications at home against a medication list obtained from the pharmacy prior to the eVisits. The scheduling of eVisits differs across physicians: From sprinkling the eVisits in 10-minute slots during their week to scheduling them for an entire afternoon. In the Epilepsy clinic, the eVisit follow-ups have replaced an entire in-person follow-up clinic for two physicians (LBL, GS). Clinic space that was freed up by the eVisits was allocated to other physicians in need of clinic space. The high uptake in these clinics is due to multiple factors including the nature of the disease, patient barriers to accessing outpatient care (lack of driving privileges, physical disability, etc.), as well as the limited requirement for detailed hands-on neurological examination during follow-ups for epilepsy and sleep. We are also using the platform to reduce inter-hospital transfers from a local rehabilitation facility to the outpatient clinic for follow-up appointments.^18^

## Case for virtual care

Virtual care modalities are hugely patient centric and enable physicians to identify risks and patient vulnerabilities sooner, improve treatment adherence, support behavioral and care interventions to improve speech, mobility, and arrange timely access to home care or community-based care/allied health services. Some of the other patient-related factors specific to neurological conditions like epilepsy and sleep are the driving restrictions, the limited requirement for detailed neurological examination during follow-up, ease of assessing speech, eye movements, coordination and gait through eVisit, cognitive and psychiatric co-morbidities causing frustration in waiting areas and the general hospital environment in general, privacy concerns due to accompaniment by family members for transportation needs. eVisits allow patients and providers to avoid traffic and congestion. It also allows patients to avoid adverse weather and road conditions, which is a challenge in both urban centers and remote communities, where sheer distances to travel for appointments are significant. This is particularly important for patient populations with neurological conditions, who often have driving restrictions or limitations. eVisits also allow patients the flexibility of scheduling their follow-up eVisit at a time and location convenient for them and their families. Family members are able to join the eVisit remotely, offering increased support to patients, which is particularly crucial for seniors. Overall, the eVisit model of care aligns with Picker’s principles of patient-centered care.^19^ eVisits also have the potential to improve population health by reducing barriers to care, reduce wait times, and improve access to timely care.

Physicians and other health care providers benefit from eVisit’s flexible scheduling, which allows being more productive with their time, enabling them to distribute their clinical activity to accommodate other commitments, including teaching, research, and administration.^20^ In addition to increased productivity, eVisits have the potential to address some of the significant contributors to physician burnout (work and organizational factors), which, in turn, can have consequences on patient care and health care costs.^21^ eVisit also reduces the need for admin/nursing support typically needed in the clinic setting, thus further reducing the overhead costs. Suitability of patients and their follow-up plan for eVisits should be an individualized decision made mutually by the physician and the patient to ensure safety.

Virtual care uptake has been significant in Canada and has facilitated safe, timely, and accessible ambulatory care during this COVID-19 crisis.^22^ This has resulted in capacity issues for the existing Ontario Telemedicine Network, the provincial telemedicine provider. The ministry of health has allowed health care providers to use video visits and telephone for providing ambulatory care during the COVID-19 crisis.^5^ Broadly, the virtual care platforms fall under regulated and unregulated categories and guidance on appropriate use, the disclaimer, and consent are available here.^22^ A comprehensive list of guidance on virtual care and platforms available across Canada is available here.^23^ Use of regulated platforms that meet the privacy and security standards for safeguarding personal health care information is strongly recommended. At Queen’s University/Kingston Health Sciences Center, Reacts (www.reacts.com) and OTN are being used for providing virtual care.^24^ Since the COVID-19 crisis, we have expanded the scope of eVisits to include new consultations. Patients are seen in-person only if the attending neurologists feel that a reasonable diagnosis cannot be made via eVisit, and if the clinical situations warrant an urgent/emergency consultation.

## Barriers in using eVisits for neurological follow-up

In our experience, we have faced many challenges and barriers to convert and sustain patients to receive virtual care, especially video visits. The challenges span across the spectrum of adoption regarding a new technology by patients and include the lack of access to technology (smart devices, computers), reliable internet connection, know-how of using technology, and ease of navigating the user interface of the virtual care platform. We have been using the telephone for contacting the patients that are not capable of doing virtual visits. Telephone visits are remunerated on a temporary basis in some provinces,^5^ and are ideal for follow-up visits to convey results and answer questions. The limitations of telephone calls include the inability to properly validate the patient and physician identification, lack of physical examination, and accurate medical reconciliation. Despite the limitations of telephone visits, they offer a very convenient option for many and further study of the safety, efficacy should be tested. Current clinical practice standards, regulatory standards, and physician remuneration are based around traditional forms of medical care performed through in-person interaction. Extensive guidelines and frameworks exist around these issues to guide clinicians. Yet, a similar framework for various virtual care modalities is yet to exist and is perceived as an immediate need by the Canadian Medical Association, Royal College of Physicians and Surgeons (Canada), and College of Family Physicians of Canada.^7,25,26^ Another significant barrier is physician remuneration. This, however, looks promising, given the ongoing work by the Canadian Medical Association led virtual care task force as well as new digital health policies adopted by provincial health care authorities.^7,8,25^

## Conclusion

Many of the chronic neurological diseases need long-term and regular follow-up for clinical activities like symptom management, medication titration, review of investigations, patient education, and counselling. As a community of health care providers caring for the people and families affected by neurological diseases, it is our duty and responsibility to leverage the existing technologies available to reduce the burden on the patients, families, health care system, and ultimately society. Extraordinary times require extraordinary measures. As the current COVID-19 pandemic is projected to last for a few more months, it is imperative for the neurology community to embrace virtual care to continue to provide care to patients affected by neurological conditions. The adoption of virtual care into a neurological practice at this time will ensure that timely care is provided to patients simultaneously avoiding contact with the hospitals, avoiding long wait lists in the future.
